# Unconditional and conditional analysis of epistasis between tillering QTLs based on single segment substitution lines in rice

**DOI:** 10.1038/s41598-020-73047-7

**Published:** 2020-09-28

**Authors:** Huaqian Zhou, Weifeng Yang, Shuaipeng Ma, Xin Luan, Haitao Zhu, Aimin Wang, Congling Huang, Biao Rong, Shangzhi Dong, Lijun Meng, Shaokui Wang, Guiquan Zhang, Guifu Liu

**Affiliations:** 1 Guangdong Key Lab of Plant Molecular Breeding, South China Agricultural University, Guangzhou, 510642 People’s Republic of China; 2 Agricultural Genomics Institute in Shenzhen, Chinese Academy of Agricultural Sciences, Shenzhen, 440307 People’s Republic of China; 3 Zhuhai Modern Agriculture Development Center, Zhuhai, 519000 People’s Republic of China; 4 Guangzhou Experimental Station, Chinese Academy of Tropical Agricultural Sciences, Guangzhou, 510642 People’s Republic of China

**Keywords:** Developmental biology, Genetics

## Abstract

Epistasis plays an important role in manipulating rice tiller number, but epistatic mechanism still remains a challenge. Here we showed the process of epistatic analysis between tillering QTLs. A half diallel mating scheme was conducted based on 6 single segment substitution lines and 9 dual segment pyramiding lines to allow the analysis of 4 epistatic components. Additive-additive, additive-dominance, dominance-additive, and dominance-dominance epistatic effects were estimated at 9 stages of development via unconditional QTL analysis simultaneously. Unconditional QTL effect (QTL cumulative effect before a certain stage) was then divided into several conditional QTL components (QTL net effect in a certain time interval). The results indicated that epistatic interaction was prevalent, all QTL pairs harboring epistasis and one QTL always interacting with other QTLs in various component ways. Epistatic effects were dynamic, occurring mostly within 14d and 21–35d after transplant and exhibited mainly negative effects. The genetic and developmental mechanism on several tillering QTLs was further realized and perhaps was useful for molecular pyramiding breeding and heterosis utilization for improving plant architecture.

## Introduction

Breeding for ideotypes implies to take qualified morphological and physiological characteristics to concentrate on one plant so as to obtain the highest utilization rate and transformation rate of light energy^1–3^. Tiller number in cereal is not only a plant type trait but also one of the important agronomic traits for grain production, and moreover a model trait for the study of developmental behaviors^4,5^. Therefore, the understanding tillering genetic mechanism will address the fundamental issues of plant science and facilitate the breeding of high-yielding crop varieties.

Using molecular marker maps and QTL mapping technology, mapping of QTLs for tiller number has been extensively conducted^6–9^. Two common methods were used for developmental traits, one being by analyzing the performance of a trait observed at a fixed stage of ontogenesis to estimate QTL accumulated effects from the beginning to the investigation stage, and the other being analyzing successively the observations at various developmental stages to reveal QTL expression dynamics^8^. However net expression of QTLs at a certain stage kept mysterious.

Understanding gene expression is one of the major goals in developmental genetics. A separate analysis of data derived from times *t-1* and *t* can provide inferences for the cumulative gene effects from the initial time to *t-1* or *t* but not for the net effect of gene expression in the period *t-1* to *t*^10^. A conditional analysis method was proposed to estimate the extra genetic effect from *t-1* to *t*^10,11^, and QTL analysis on the extra genetic effect (named conditional QTL analysis) had widely been applied^12–15^. Conditional QTL reflected the net expression of QTL at a certain time. However traditional QTL mapping can not obtain an unbiased estimation of QTLs under the conventional mapping populations such as F2, RILs, or DHLs.

It has been long to recognize the advantages of near isogenic lines or single segment substitution lines for QTL identification^16–18^. Based on single segment substitution lines numerous QTLs were identified^19–32^. However, the important genetic component of epistasis was often ignored or incomprehensive. Japanese rice genome projects (JRGP) and Guangdong Key Lab of Plant Molecular Breeding provided typical cases to analyze epistatic interactions between QTLs, in which four types of epistatic components were effectively estimated on heading date in rice via secondary mapping populations^33–37^. In this paper, we applied previous cultured materials to estimate epistasis between QTLs on tiller number via adopting both the unconditional and conditional analysis methods. The aims were to further confirm the prevalence of epistatic interactions and deeply understand genetic and developmental mechanisms on several tillering QTLs so as to excavate useful alleles for molecular pyramiding breeding and heterosis utilization for improving plant architecture.

## Results

### Identification of tillering QTLs

Tiller number appeared “S” type of growth curve, after slow, rapid growth it reaching the peak and then decreasing slightly (Supplementary Figure S1). Analysis of variance at various stages revealed the significant difference of tiller numbers existed among genotypes (Supplementary Table S1). This information supported the existence of tillering QTLs. The contrast test indicated that the 6 SSSLs really harbored tillering QTLs (Table 1). All carried with heterozygote effects, and S_2_ with additional homozygote effects. That QTLs appeared repeatedly at several tested stages guaranteed the truth of putative QTLs, but inconsistent estimations throughout stages exhibited dynamics of QTL expressions. The effects of QTLs were mainly to enhance tillering and appeared mostly before *t3*. It was suggested that utilization of these QTLs on heterosis and in developmental early stages.

**Table 1:** Homozygote and heterozygote effects of SSSLs estimated on tiller numbers at various developmental stages.

SSSL	Effect	*t1*	*t2*	*t3*	*t4*	*t5*	*t6*	*t7*	*t8*	*t9*
S_1_	*a*	− 0.06	0.22	− 0.11	− 1.33	− 2.08	− 2.00	− 2.17	− 2.00	− 1.08
	*d*	0.78*	0.78	1.50*	− 0.17	− 1.67	− 1.00	− 1.67	− 1.08	− 0.25
S_2_	*a*	0.50	0.94*	1.33*	1.42	0.08	0.50	0.25	− 0.08	− 0.17
	*d*	1.33**	2.72**	3.81**	2.83*	1.00	− 0.54	0.17	0.00	0.04
S_3_	*a*	0.17	0.28	0.39	0.42	0.25	1.75	1.75	2.17	1.75
	*d*	0.39	0.83	1.44*	2.00	3.75*	4.42**	4.25**	4.25**	4.58**
S_4_	*a*	0.00	0.06	0.22	0.00	0.25	1.67	1.08	1.00	1.08
	*d*	0.61	1.44**	2.33**	0.67	− 0.58	− 0.25	− 0.67	− 0.83	− 0.67
S_5_	*a*	0.06	0.22	0.50	0.42	0.42	0.83	0.92	0.67	1.00
	*d*	0.94**	1.78**	2.22**	2.08	1.50	2.00	2.08	1.33	1.92
S_6_	*a*	0.06	− 0.28	− 0.22	− 0.83	− 1.33	− 1.67	− 1.64	− 1.42	− 1.32
	*d*	0.89*	1.17*	1.67*	0.67	0.08	− 0.17	− 0.17	− 0.25	0.00

SSSL was the abbreviation of single segment substitution line. S_i_ represented the code of SSSL_i_. *ti* indicated various developmental stages, the difference of 7d. *a* and *d* were homozygote and heterozygote effects, respectively, estimated by $SSSL_{i} - HJX74$(where *i* represented homozygote or heterozygote). Sign “− ” meant to descend tiller number due to the alleles from donors. Superscripts “^*^ and ^**^” indicated the significance at 5% and 1% level, respectively.

### Estimations of epistatic effects

The epistatic effect was generally estimated as the deviation between the dual-SSSL pyramiding effect and the sum of two single-SSSL effects. Estimations of four epistatic components, additive-additive (*aa*), additive-dominance (*ad*), dominance-additive (*da*), and dominance-dominance (*dd*) for nine pairs of QTLs confirmed epistatic prevalence (Table 2). All pairs of S_i_/S_j_ held interaction effects, involving in one or more epistatic components and exhibiting at least two stages. Interactions in *dd* component, with negative effects and at early stages were in the majority, which closely related to *d* component, positive effects and early development of single QTL. Consistent function directions appeared among epistatic components and across stages. However, epistasis changed with developmental stages, reflecting dynamics of it. Additionally, one QTL always interacted with multiple other QTLs in various component ways.

**Table 2:** Epistatic effects between QTLs estimated on tiller number at various developmental stages.

SSSL combination	Effect	*t1*	*t2*	*t3*	*t4*	*t5*	*t6*	*t7*	*t8*	*t9*
S_1_/S_2_	*aa*	− 0.61	− 1.61*	− 1.78*	− 1.75	0.25	− 0.42	0.25	0.50	0.58
	*ad*	− 0.58	− 3.29**	− 3.91**	− 0.67	1.03	2.85	2.89	3.36	3.21
	*da*	− 0.94*	− 0.89	− 1.78*	− 1.17	1.83	0.92	1.25	1.50	1.33
	*dd*	− 1.94**	− 3.56**	− 5.14**	− 1.67	0.42	1.79	1.58	1.08	1.04
S_1_/S_3_	*aa*	− 0.11	− 0.67	− 0.61	0.17	0.58	0.08	0.50	− 0.17	− 0.17
	*ad*	0.33	− 1.06	− 0.72	− 0.33	− 1.67	− 2.25	− 2.25	− 2.08	− 2.67
	*da*	− 0.94*	− 1.22*	− 1.83*	− 1.33	− 0.67	− 2.00	− 1.17	− 1.83	− 2.00
	*dd*	− 1.17*	− 1.50*	− 2.89**	− 1.67	− 3.75*	− 5.00*	− 3.75*	− 4.22**	− 5.00**
S_1_/S_4_	*aa*	0.06	− 0.56	− 0.39	0.08	− 0.92	− 2.42	− 1.50	− 1.08	− 1.83
	*ad*	0.06	− 1.50*	− 1.56*	1.42	2.42	2.75	2.97	3.28	3.25
	*da*	0.00	0.39	1.11	3.17	5.17*	5.25**	6.75**	6.33**	5.75**
	*dd*	− 1.17*	− 2.56**	− 4.11**	− 1.33	0.67	− 0.92	0.50	0.25	− 0.33
S_1_/S_5_	*aa*	0.00	− 0.94	− 0.94	0.83	0.58	0.25	0.75	1.08	0.17
	*ad*	− 0.28	− 1.78*	− 1.19	1.25	2.00	2.25	2.11	2.86	2.72
	*da*	− 0.50	− 0.67	− 1.33	− 0.58	0.25	− 1.33	− 0.50	− 0.83	− 1.83
	*dd*	− 1.50**	− 2.72**	− 3.78**	− 1.67	− 1.33	− 2.17	− 0.58	0.08	− 0.83
S_2_/S_3_	*aa*	− 0.72	− 1.28*	− 1.67*	− 2.75	− 0.58	− 2.83	− 2.42	− 2.75	− 2.00
	*ad*	− 0.44	− 1.00	− 1.28	− 1.92	− 3.83*	− 4.42*	− 4.00*	− 3.50*	− 4.00**
	*da*	− 1.50**	− 2.94**	− 3.69**	− 3.00	− 1.75	− 2.29	− 2.17	− 2.42	− 1.79
	*dd*	− 0.83	− 1.94**	− 1.81*	− 0.25	1.17	3.79*	3.58*	3.44*	2.93*
S_2_/S_4_	*aa*	− 0.61	− 1.33*	− 1.72*	− 2.83	− 1.83	− 3.75	− 2.75	− 2.17	− 2.08
	*ad*	− 1.17*	− 2.39**	− 3.17**	− 1.58	0.92	0.83	1.67	1.92	1.42
	*da*	− 1.50**	− 3.06**	− 3.81**	− 1.67	− 1.00	− 0.63	− 0.67	− 0.67	− 0.88
	*dd*	− 2.00**	− 4.56**	− 6.19**	− 4.00*	− 2.25	0.04	0.17	0.50	0.46
S_2_/S_5_	*aa*	− 0.72	− 1.33*	− 2.00*	− 2.75	− 1.25	− 1.58	− 1.42	− 0.50	− 0.58
	*ad*	− 1.50**	− 3.94**	− 4.61**	− 4.50*	− 3.67*	− 4.50*	− 3.83*	− 2.67	− 2.83
	*da*	− 1.44**	− 3.22**	− 4.47**	− 3.58*	− 2.25	− 0.46	− 1.58	− 1.00	− 1.29
	*dd*	− 2.39**	− 5.39**	− 6.86**	− 5.92**	− 3.83*	− 3.04	− 3.83*	− 2.58	− 2.79
S_3_/S_6_	*aa*	− 0.11	0.50	0.33	0.58	0.92	− 0.33	− 0.28	− 1.58	− 1.00
	*ad*	− 0.72	− 0.94	− 1.06	0.08	− 0.83	− 1.83	− 2.00	− 2.92	− 2.42
	*da*	0.61	1.06	1.28	0.50	− 0.42	− 0.58	− 0.19	− 0.67	− 0.83
	*dd*	− 0.78	− 2.33**	− 2.61**	− 1.75	− 4.00*	− 5.17*	− 4.08*	− 4.42*	− 5.00**
S_4_/S_6_	*aa*	− 0.11	− 0.22	0.00	0.17	0.08	0.42	1.06	1.17	0.75
	*ad*	− 1.06*	− 1.44*	− 2.11*	− 0.92	− 0.83	− 1.08	− 0.17	− 0.75	− 0.67
	*da*	− 0.83	− 1.67**	− 2.39**	0.17	2.17	2.33	2.47	2.67	2.67
	*dd*	− 1.44**	− 2.61**	− 3.89**	− 0.92	− 1.00	− 0.25	− 0.17	− 0.08	− 0.67

SSSL was the abbreviation of single segment substitution line. S_i_ represented the code of SSSL_i_*. ti* indicated various developmental stages, the difference of 7d. *aa, ad, da* and *dd* were additive-additive, additive-dominance, dominance-additive and dominance-dominance epistasis respectively, estimated by $(D_{kl} + HJX74 - S_{k} - S_{l} )$, where $D_{kl} ,S_{k} ,S_{l}$ indicated dual segment and its two single segment materials respectively, which might be homozygotes or heterozygotes. Sign ”-” meant to descend tiller number due to the alleles from donors. Superscripts “^*^ and ^**^” indicated the significance at 5% and 1% level, respectively.

### Conditional QTL analysis

Unconditional QTL analysis mentioned above indicated that tillering QTLs changed dynamically (Tables 1, 2). Conditional QTLs revealed expression quantities of tillering QTLs in various time intervals (Table 3). For instance, the cumulative heterozygote effect of S_1_ was 1.50^*^ at *t3* (Table 1), which was divided into three components, 0.78^*^, − 0.26 and 0.50, representing the net expressions in *t0–t1*, *t1–t2* and *t2–t3*, respectively (Table 3). Similarly, the cumulative *ad* component of S_1_/S_2_ was − 3.91^**^ at *t3* (Table 2), which expressed − 0.58, − 2.52^**^ and 0.31 in *t0–t1, t1–t2* and *t2–t3*, respectively (Table 3). It was indicated that QTL effects estimated at *t*(*n*) were in fact the accumulation of expression quantities in various time intervals before *t*(*n*). Expression quantities provided information about both action periods and dynamic patterns of QTLs. QTLs expressed homozygote effects mainly concentrating on *t5–t6*, heterozygote effects in *t0–t1* and *t3–t6,* while epistasis in *t0–t2* and *t3–t5*. Great differences took place among epistatic components and developmental periods. Negative expressions were main, occupied up 66.7% of all significant effects, where negative heterozygote effects and negative epistatic effects hold 47.1% and 75.7%, respectively. Inverse correlation between heterozygote effects and epistatic effects was detected in *t0–t2* and *t3–t5*, where positive (negative) *d* resulting in negative (positive) *e* in general. Lots of expressions were feeble so that fail to be detected, and large expressions became invisible for the reason of error ascending.

**Table 3:** Conditional QTL effects on tiller number estimated in various developmental time intervals.

SSSLand their combination	Effect	*t1*	*t2|t1*	*t3|t2*	*t4|t3*	*t5|t4*	*t6|t5*	*t7|t6*	*t8|t7*	*t9|t8*
S_1_	*a*	− 0.06	0.30	− 0.40	− 1.21	− 0.59	− 0.44	− 0.35	0.06	0.76
	*d*	0.78*	− 0.26	0.50	− 1.77*	− 1.48*	0.25	− 0.76	0.50	0.75
S_2_	*a*	0.50	0.28	0.12	− 0.01	− 1.50*	0.44	− 0.21	− 0.32	− 0.09
	*d*	1.33**	0.95*	0.32	− 1.24*	− 2.17*	− 1.29*	0.66	− 0.16	0.04
S_3_	*a*	0.17	0.06	0.03	0	− 0.22	1.56*	0.16	0.50	− 0.24
	*d*	0.39	0.32	0.38	0.45	1.51*	1.60*	0.23	0.21	0.67
S_4_	*a*	0	0.06	0.15	− 0.24	0.25	1.48*	− 0.43	− 0.03	0.16
	*d*	0.61	0.63	0.48	− 1.83*	− 1.33*	0.19	− 0.44	− 0.20	0.10
S_5_	*a*	0.06	0.15	0.22	− 0.12	− 0.05	0.52	0.16	− 0.20	0.39
	*d*	0.94**	0.52	− 0.05	− 0.29	− 0.83	0.88	0.26	− 0.65	0.69
S_6_	*a*	0.06	− 0.35	0.13	− 0.60	− 0.40	− 0.67	− 0.12	0.14	0.14
	*d*	0.89*	− 0.02	0.17	− 1.12	− 0.66	− 0.23	− 0.02	− 0.09	0.23
S_1_/S_2_	*aa*	− 0.61	− 0.80	0.28	0.15	2.21	− 0.60	0.63	0.26	0.12
	*ad*	− 0.58	− 2.52**	0.31	3.52*	1.77	2.08	0.30	0.62	0.12
	*da*	− 0.94	0.37	− 0.64	0.74	3.14*	− 0.46	0.42	0.31	− 0.05
	*dd*	− 1.94	− 0.93	− 0.59	3.83**	2.28	1.48	− 0.05	− 0.42	0.04
S_1_/S_3_	*aa*	− 0.11	− 0.52	0.24	0.82	0.40	− 0.35	0.42	− 0.64	− 0.01
	*ad*	0.33	− 1.50**	0.63	0.44	− 1.29	− 1.00	− 0.20	0.05	− 0.75
	*da*	− 0.94	0.03	− 0.27	0.63	0.83	− 1.50	0.65	− 0.72	− 0.31
	*dd*	− 1.17	− 0.21	− 0.46	− 0.35	− 3.36*	− 1.94	0.04	− 0.16	− 0.37
S_1_/S_4_	*aa*	0.06	− 0.63	0.32	0.50	− 1.01	− 1.73	0.70	0.34	− 0.84
	*ad*	0.06	− 1.57**	0.36	3.08*	0.83	0.94	0.47	0.45	0.23
	*da*	0	0.39	0.61	1.98	1.62	1.38	1.97	− 0.08	− 0.08
	*dd*	− 1.17*	− 1.00*	− 0.84	3.07*	2.16	− 1.42	1.33	− 0.22	− 0.56
S_1_/S_5_	*aa*	0	− 0.94	0.26	1.84	− 0.35	− 0.19	0.52	0.37	− 0.83
	*ad*	− 0.28	− 1.41*	1.09*	2.52	0.60	0.75	0.06	0.86	0.09
	*da*	− 0.50	0	− 0.48	0.84	0.90	− 1.52	0.71	− 0.36	− 1.07
	*dd*	− 1.50**	− 0.73	− 0.29	2.38	0.53	− 1.17	1.39	0.64	− 0.91
S_2_/S_3_	*aa*	− 0.72	− 0.15	0.35	− 0.10	0.04	− 1.50	0.09	0.01	0.14
	*ad*	− 0.44	− 0.41	0	− 0.55	− 1.69	− 1.54	0.02	0.30	− 0.78
	*da*	− 1.50**	− 0.95*	0.07	0.95	1.61	− 0.98	− 0.08	− 0.36	0.43
	*dd*	− 0.83	− 0.84	0.68	1.68	1.45	2.92	0.13	0.04	− 0.24
S_2_/S_4_	*aa*	− 0.61	− 0.26	0.22	0.21	1.11	− 1.15	0.90	0.16	− 0.41
	*ad*	− 1.17*	− 0.84	− 0.11	1.81	2.69*	0.15	0.91	0.33	− 0.35
	*da*	− 1.50**	− 1.06*	0.11	2.41	0.87	0.12	− 0.10	− 0.03	− 0.26
	*dd*	− 2.00**	− 1.90**	− 0.36	2.63	2.23	1.73	0.13	0.34	0
S_2_/S_5_	*aa*	− 0.72	− 0.37	− 0.29	− 0.61	1.83	− 0.65	0.02	0.85	− 0.12
	*ad*	− 1.50**	− 1.95**	0.44	0.43	1.37	− 1.75	0.26	0.98	− 0.38
	*da*	− 1.44**	− 1.30*	− 0.35	1.2	1.76	1.23	− 1.17	0.50	− 0.37
	*dd*	− 2.39**	− 2.21**	0.04	1.42	2.79*	− 0.17	− 1.07	1.06	− 0.41
S_3_/S_6_	*aa*	− 0.11	0.35	0.19	0.69	− 1.19	− 0.77	− 0.22	− 1.25	0.36
	*ad*	− 0.72	0.02	0.15	1.21	− 0.93	− 1.21	− 0.33	− 1.02	0.27
	*da*	0.61	0.24	− 0.07	− 0.87	− 0.98	− 0.27	0.34	− 0.48	− 0.22
	*dd*	− 0.78	− 1.30*	0.38	1.04	− 2.04	− 2.17	0.62	− 0.54	− 0.94
S_4_/S_6_	*aa*	− 0.11	− 0.07	0.28	0.17	− 0.10	0.35	0.68	0.16	− 0.32
	*ad*	− 1.06*	− 0.04	− 0.26	1.34	0.19	− 0.46	0.82	− 0.59	0.02
	*da*	− 0.83	− 0.56	− 0.26	2.72	1.98	0.71	0.35	0.32	0.21
	*dd*	− 1.44**	− 0.69	− 0.55	3.24*	0.03	0.50	0.06	0.08	− 0.59

SSSL was the abbreviation of single segment substitution line. *S*_*i*_ represented the code of SSSL_i_. *t(n)|t(n-1)* indicated the developmental stage from *n-1* to *n*, 7d. *a* and *d* were homozygote and heterozygote effects respectively, estimated by $SSSL_{i} - HJX74$(where *i* represented homozygote or heterozygote). *aa, ad, da* and *dd* were additive-additive, additive-dominance, dominance-additive and dominance-dominance epistasis, respectively, estimated by $(D_{kl} + HJX74 - S_{k} - S_{l} )$, where $D_{kl} ,S_{k} ,S_{l}$ indicated dual segment and its two single segment materials respectively, which might be homozygotes or heterozygotes. Sign “-” meant to descend tiller number due to the alleles from donors. Superscripts “^*^ and ^**^” indicated the significance at 5% and 1% level, respectively.

## Discussion

Tiller number related to plant architecture and yield components. Generally, tiller number was investigated at the maximum tillering stage and was then analyzed. This however didn’t provide ample information about QTLs. QTLs detected at *t6* (the maximum tillering stage, see Supplementary Figure S1) were significantly less than those at *t2* or *t3* (Tables 1, 2). It is suggested that an alternative strategy to explore tillering QTLs concentrating in 14–21d after transplant. QTL analysis of time-specific measures possessed great advantages, providing comprehensive and convincing QTL information (Tables 1, 2). Especially time-specific measures allowed to excavate QTL dynamics. Conditional QTL analysis estimated net expression quantities of tillering QTLs in various developmental time intervals (Table 3), solving the puzzle of gene dynamic behavior. Since SSSLs harbored tillering QTLs mostly with positive homozygote effects and heterozygote effects (Table 1), negative epistatic effects were detected between many SSSL pairs (Table 2). QTLs expressed selectively in certain time intervals, homozygote effects appearing mostly in 35–42d, heterozygote effects in 0–7d and 21–42d, and epistasis in 0–7d and 21–35d after transplant (Table 3).

Unconditional QTL and conditional QTL analysis produced approximate consistent results (Tables 1–3). In theory, the unconditional QTL effect is the accumulation of several conditional QTL components^10^. Generally, QTL effects at time *t*_*n*_ are the sum of QTL effect at time *t*_*n-1*_ and the extra effect *G*_*d*_. *G*_*d*_ was ever estimated by the deviation of QTL effects between *t*_*n*_ and *t*_*n-1*_, but it couldn’t provide the net effects of gene expression in the period *t*_*n-1*_ to *t*_*n*_ since *G*_*d*_ correlated with QTL effect at *t*_*n-1*_^10,11^. The conditional QTL effect was as the unbiased estimation of *G*_*d*_, namely $QTL_{{t_{n} }} = QTL_{{t_{n - 1} }} + QTL_{{t_{n} |t_{n - 1} }}$ since $QTL_{{t_{n} |t_{n - 1} }}$ was independent of $QTL_{{t_{n - 1} }}$^12–15^. The formula could be extended easily as $QTL_{{t_{n} }} = QTL_{{t_{1} }} + QTL_{{t_{2} |t_{1} }} + QTL_{{t_{3} |t_{2} }} + \cdots + QTL_{{t_{n} |t_{n - 1} }}$. The relationship was well validated by estimated results (Supplementary Table S2), in which the correlation coefficient between $QTL_{{t_{9} }}$ effects and the sums of all conditional QTL effects before t9 reached 0.9379^**^. Furthermore, only did conditional QTL variations reflected dynamic expressions of a QTL throughout the whole developmental stage.

In multiple gene system, gene interaction is inevitable except for gene accumulative effects^11^. A half diallel mating design allowed to estimate various components simultaneously, time-specific measures provided repeated testing, and conditional analysis revealed net expressions of epistasis (Table 2). All SSSL pairs involved in *dd* and another epistatic component at least, with 90.6% negative estimations and 65.6% of them detected in 21d after transplant (Table 2). QTLs expressed epistasis mostly with negative effects (occupied 75.7%) and mainly in 14d and 21–35d after transplant (Table 3). These epistatic features attributed to the inverse correlation between heterozygote effect and epistasis. Additionally, expression quantity differences exhibited abundantly the dynamics of epistasis with the development of tillers.

Tillering QTLs were widely located via traditional mapping populations such as F2, DHLs and RILs etc^6–9^. However, since both the materials and markers applied in these studies differed, it was difficult to compare the locations of existing QTLs on tiller number. Our lab was engaged in the study of QTLs using the population of single segment substitution lines for a long time, it was possible to detect the same chromosome regions for different QTLs. In 2008, we identified 18 QTLs on panicle number in rice using SSSLs^38^, and then nine of them were analyzed for their dynamic expression in 2009^30^. Similarly, 14 tillering QTLs were detected with a single segment substitution population of 26 lines by unconditional QTL mapping method, and their net expressions were estimated within time intervals by conditional QTL mapping simultaneously in 2010^31^, and then twelve of them were analyzed for their dynamic expression in different planting seasons and under different cropping densities in 2012^32^. These studies excavated tillering QTLs and showed dynamic expressions for some of them on additive, dominance and dominance-dominance epistatic component, but lacking the components of additive-additive, additive-dominance and dominance-additive epistatic components since the limitation of pyramiding line materials. In 2018 based on eight single segment substitution lines, each of which was tested with heading QTLs, a half diallel crossing population was constructed to allow estimating simultaneously the four epistatic components of QTLs^37^. Using this population, six single segment substitution lines were tested to be with tillering QTLs, and then their dynamic expressions of the four epistatic components at various developmental stages were explored in this paper. Comparing with the previous studies, the chromosome interval of S_1_ in this paper might be consistent with SSSL 23 in 2008, S_2_ with SSSL 14 in 2008, *Tn6-2, Tn6-3* and *Tn6-4* in 2010, and S_3_ with SSSLs 3, 14, 22 in 2008, *Tn6-2, Tn6-3, Tn6-4* in 2010 (Supplementary Figure S2) .

## Methods

### Plant materials

The similar plant materials as the previous experiment were applied in this trial^37^ (Table 4). Huajingxian 74 (HJX74) is an elite *indica* variety with many excellent properties cultured by our laboratory from South China. SSSLs possessed only a single substituted segment from donors under HJX74 genetic background, and harbored putative QTL/gene on heading date^37^. Double segment substitution lines (DSSLs) were cultivated based on the F2 populations from the crossing of two SSSLs. Together with of 6 SSSLs, a total of nine DSSLs were selected as parental materials (Table 2), Some of DSSLs were lacking since their seeds of F1 weren’t achieved. And then the experimental population was compounded via a half diallel mating scheme37. The population in total included 49 materials, HJX74, 6 SSSLs, 9 DSSLs and 33 crossing combinations among them.

**Table 4:** Single segment substitution lines (SSSLs) and their basic information.

SSSL	Code	Chr	Marker on subsitituted segment	Donor parent	The length of substitution segments (cM)
W23-03–08-09–27-82	S_1_	3	End–PSM301-PSM304–RM569	Lemont	7.9
W08-18–09-09–06-02	S_2_	6	RM549-RM136-RM527	IR64	18.0
W04-47–68-05–04-04–02-02	S_3_	6	RM510–RM204–RM50–RM549	BG367	26.8
W06-26–35-01–05-02	S_4_	8	PSM152–PSM154-RM72–RM404	Katy	20.4
W11-17–03-07–05-08	S_5_	10	PSM166–RM596-RM271–RM269	Basmati 370	16.7
W27-18–03-21	S_6_	10	RM467–PSM166-RM304–RM294A	IAPAR9	32.9

SSSL was the abbreviation of single segment substitution line. S_i_ represented the code of SSSL_i_. Chr was the abbreviation of chromosome.

### Field experiments

The same phenotypic trial as the previous study was applied in this trial^37^. The trial site located at the teaching and experiment station of South China Agricultural University, in Guangzhou, China (23°79ʹ N, 113°159ʹ E). In the early season (duration from March to July) of 2018, 49 materials were grown in a completely randomized block design with three replications. Germinated seeds were sowed in a seedling bed, and then seedlings were transplanted to a rice field 20 days later with one plant per hill and the density of 16.7 cm $\times$ 16.7 cm. A plot consisted of four rows, ten plants per row. Local standard practices were used for the trails. Tiller numbers per hill on 10 central plants were investigated in each plot from seven days after transplanting onwards, data every 7 days once was continuously recorded nine weeks (denoted by *t1* to *t9*). Averages over ten plants in each plot were as inputting data for statistical analysis.

### Statistical analysis and estimation of QTL effects

The model $y_{ij} = \mu + G_{i} + B_{j} + e_{ij}$ was adopted to variance analysis on data at each stage, where $y,\mu ,G,B$ and $e$ were the observations each plot, the population mean, genotype, block and error effect, respectively. *i*, *j* represented serial numbers of genotypes and blocks, respectively. Homozygote effect (*a*) or heterozygote effect (*d*) of QTL was estimated by $(S_{i} - HJX74)$, where $S_{i}$ represented homozygote or heterozygote of SSSL_*i*_. Epistatic effect between QTLs (*e*) was estimated by $(D_{kl} + HJX74 - S_{k} - S_{l} )$, where $D_{kl} ,S_{k} ,S_{l}$ indicated dual segment and its two single segment materials, respectively, which might be homozygotes or heterozygotes. Four epistatic components could be estimated by,

$$\begin{gathered} aa = D_{ij} + HJX74 - S_{i} - S_{j} \hfill \\ ad = D_{ij^{\prime}} + HJX74 - S_{i} - S_{j^{\prime}} \hfill \\ da = D_{i^{\prime}j} + HJX74 - S_{i^{\prime}} - S_{j} \hfill \\ dd = D_{i^{\prime}j^{\prime}} + HJX74 - S_{i^{\prime}} - S_{j^{\prime}} \hfill \\ \end{gathered}$$

where $aa,ad,da,dd$ indicated additive-additive, additive-dominance, dominance-additive and dominance-dominance epistatic components, respectively. $k = i, \, i^{\prime}, \, l = j, \, j^{\prime}$, *i* and *i′*, *j* and *j′* represented homozygotes and heterozygotes of substitution segments. Statistics $t_{\alpha } \sqrt {\frac{{2S_{e}^{2} }}{n}}$ and $t_{\alpha } \sqrt {\frac{{4S_{e}^{2} }}{n}}$ were adopted to test of significance for *a/d* and *e*, respectively. Where $t_{\alpha }$, $S_{e}^{2}$, $n$ indicated as critical *t*-value under $\alpha$ probability level and error freedom degree, error mean square and replication numbers, respectively. Formula $y_{t|t - 1} = y_{t} - b_{t/t - 1} (y_{t - 1} - \overline{y}_{t - 1} )$ was used to estimate conditional variable $y_{t|t - 1}$, indicating phenotypic values at time* t* conditional on the phenotypic value at given time *t-1,* where $y_{t - 1} {\text{ and }} \overline{y}_{t - 1} , \, y_{t} {\text{ and }} \overline{y}_{t}$ were the phenotypic values and means at time *t-1* and time *t*, respectively. $b_{t/t - 1}$ was the regression coefficient for phenotypic values at time *t* versus time *t-1*. Statistical analysis was imposed on the conditional variable $y_{t|t - 1}$ to generate conditional QTLs^10^.

Statistical analysis and estimation of QTL effects were carried out with aov() and lm() functions in R language (https://www.r-project.org/).

## Supplementary information

Supplementary file1

Supplementary file2

Supplementary file3
