# Supplementary material for: Unconditional and conditional analysis of epistasis between tillering QTLs based on single segment substitution lines in rice
Source: Sci Rep. 2020 Sep 28;10:15912. doi: 10.1038/s41598-020-73047-7 (PMC7523009; doi:10.1038/s41598-020-73047-7)

Unconditional and conditional analysis of epistasis between tillering QTLs based on single segment substitution lines in rice

Huaqian Zhou^1#^, Weifeng Yang^1#^, Shuaipeng Ma^1,4#^, Xin Luan^1^, Haitao Zhu^1^, Aimin Wang^3^, Congling Huang^3^, Biao Rong^3^, Shangzhi Dong^3^, Lijun Meng^2*^ Shaokui Wang^1*^, Guiquan Zhang^1*^ Guifu Liu^1,3*^

Figure Legend

Supplementary Figure 1 Dynamics of tiller number at various developmental stages. *t1, t2, ..., t9* indicated various developmental stages.


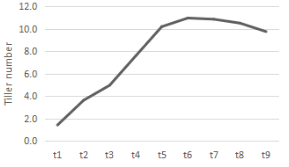

Supplement: Supplementary file 1 — Supplementary file1 [file 41598_2020_73047_MOESM1_ESM.docx]
