# Supplementary material for: Unconditional and conditional analysis of epistasis between tillering QTLs based on single segment substitution lines in rice
Source: Sci Rep. 2020 Sep 28;10:15912. doi: 10.1038/s41598-020-73047-7 (PMC7523009; doi:10.1038/s41598-020-73047-7)

Unconditional and conditional analysis of epistasis between tillering QTLs based on single segment substitution lines in rice

Huaqian Zhou^1#^, Weifeng Yang^1#^, Shuaipeng Ma^1,4#^, Xin Luan^1^, Haitao Zhu^1^, Aimin Wang^3^, Congling Huang^3^, Biao Rong^3^, Shangzhi Dong^3^, Lijun Meng^2*^ Shaokui Wang^1*^, Guiquan Zhang^1*^ Guifu Liu^1,3*^

Figure Legend

Supplementary Figure 2 The chromosome regions of tillering QTLs detected by single segment substitution lines (generated by the software MapChart 2.32, <https://www.wur.nl/en/show/Mapchart.htm>). chr was the abbreviation of chromosome, followed by a chromosome number. Vertical line represented the length of substitution segment on single segment substitution line with the right number, where the number with * was applied in the paper in 2008, *Tn* chromosome number-QTL number in 2010, and S_i_ in this paper. Comparing found that S_1_, S_2_ and S_3_ in this paper had the same chromosome regions with other single segment substitution lines applied in the previous studies.


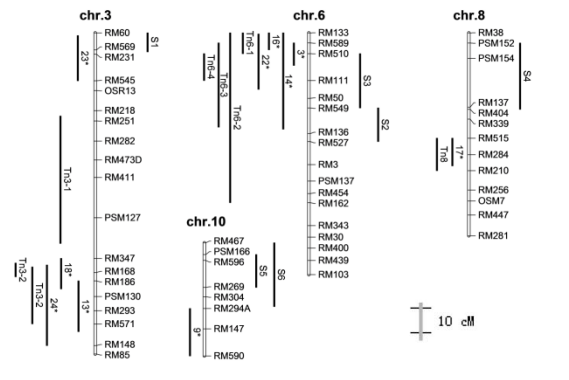

Supplement: Supplementary file 2 — Supplementary file2 [file 41598_2020_73047_MOESM2_ESM.docx]
