# Supplementary material for: Unconditional and conditional analysis of epistasis between tillering QTLs based on single segment substitution lines in rice
Source: Sci Rep. 2020 Sep 28;10:15912. doi: 10.1038/s41598-020-73047-7 (PMC7523009; doi:10.1038/s41598-020-73047-7)
Supplement: Supplementary file 3 — Supplementary file3 [file 41598_2020_73047_MOESM3_ESM.docx]

Unconditional and conditional analysis of epistasis between tillering QTLs based on single segment substitution lines in rice

Huaqian Zhou^1#^, Weifeng Yang^1#^, Shuaipeng Ma^1,4#^, Xin Luan^1^, Haitao Zhu^1^, Aimin Wang^3^, Congling Huang^3^, Biao Rong^3^, Shangzhi Dong^3^, Lijun Meng^2*^ Shaokui Wang^1*^, Guiquan Zhang^1*^ Guifu Liu^1,3*^

Supplementary Table 1 Analysis of variance on tiller number data at various developmental stage. *SST, SSg and SSe* represented the total, genotype and error [sum](E:/Program%20Files/Youdao/Dict/7.5.2.0/resultui/dict/?keyword=sum)[of](E:/Program%20Files/Youdao/Dict/7.5.2.0/resultui/dict/?keyword=of)[squares](E:/Program%20Files/Youdao/Dict/7.5.2.0/resultui/dict/?keyword=squares), respectively. *MS* was the error mean square with the freedom degree of 98. *F-value* was with the freedom degree of (48,98). *FDIST* was the significant probability of F-test. If *FDIST* was less than 0.05, then the difference among genotypic values was significant. *ti* indicated various developmental stages, the difference of 7d.

| Item | *t1* | *t2* | *t3* | *t4* | *t5* | *t6* | *t7* | *t8* | *t9* |
| --- | --- | --- | --- | --- | --- | --- | --- | --- | --- |
| *SST* | 39.43 | 120.75 | 230.70 | 495.18 | 949.13 | 883.80 | 802.11 | 772.03 | 730.00 |
| *SSg* | 21.22 | 81.22 | 165.73 | 227.89 | 368.16 | 544.06 | 534.26 | 498.34 | 494.03 |
| *SSe* | 18.21 | 39.54 | 64.97 | 267.29 | 580.98 | 339.74 | 267.85 | 273.69 | 235.97 |
| *MS* | 0.19 | 0.40 | 0.66 | 2.73 | 5.93 | 3.47 | 2.73 | 2.79 | 2.41 |
| *F-value* | 2.38 | 4.19 | 5.21 | 1.74 | 1.29 | 3.27 | 4.07 | 3.72 | 4.27 |
| *FDIST* | 0.0001 | 0.0000 | 0.0000 | 0.0106 | 0.1417 | 0.0000 | 0.0000 | 0.0000 | 0.0000 |

Supplementary Table 2 QTL effects at stage *t9* and the sum of conditional QTL components in various time intervals before *t9.* SSSL was the abbreviation of single segment substitution line. *S_i_* represented the code of SSSL_i_. *a* and *d* were additive effect and dominant effect respectively, estimated by $SSSL_{i}-HJX74$(where *i* represented homozygote or heterozygote). *aa, ad, da* and *dd* were additive-additive, additive-dominance, dominance-additive and dominance-dominance epistasis, respectively, estimated by$(D_{kl}+HJX74-S_{k}-S_{l})$, where $D_{kl},S_{k},S_{l}$indicated dual segment and its two single segment materials respectively, which might be homozygotes or heterozygotes. *t9* indicated the final stage. Sum is the sum of conditional effect. Sign “-” meant to descend tiller number due to the alleles from donors.

| SSSL | Effect | *t9* | Sum | SSSL | Effect | *t9* | Sum |
| --- | --- | --- | --- | --- | --- | --- | --- |
| S_1_ | *a* | -1.08 | -1.93 | S_1_/S_5_ | *aa* | 0.17 | 0.68 |
|  | *d* | -0.25 | -1.49 |  | *ad* | 2.72 | 4.28 |
| S_2_ | *a* | -0.17 | -0.79 |  | *da* | -1.83 | -1.48 |
|  | *d* | 0.04 | -1.56 |  | *dd* | -0.83 | 0.34 |
| S_3_ | *a* | 1.75 | 2.02 | S_2_/S_3_ | *aa* | -2.00 | -1.84 |
|  | *d* | 4.58 | 5.76 |  | *ad* | -4.00 | -5.09 |
| S_4_ | *a* | 1.08 | 1.40 |  | *da* | -1.79 | -0.81 |
|  | *d* | -0.67 | -1.79 |  | *dd* | 2.93 | 4.99 |
| S_5_ | *a* | 1.00 | 1.13 | S_2_/S_4_ | *aa* | -2.08 | 0.17 |
|  | *d* | 1.92 | 1.47 |  | *ad* | 1.42 | 3.42 |
| S_6_ | *a* | -1.32 | -1.67 |  | *da* | -0.88 | 0.56 |
|  | *d* | 0.00 | -0.85 |  | *dd* | 0.46 | 2.80 |
| S_1_/S_2_ | *aa* | 0.58 | 1.64 | S_2_/S_5_ | *aa* | -0.58 | -0.06 |
|  | *ad* | 3.21 | 5.62 |  | *ad* | -2.83 | -2.10 |
|  | *da* | 1.33 | 2.89 |  | *da* | -1.29 | 0.06 |
|  | *dd* | 1.04 | 3.66 |  | *dd* | -2.79 | -0.94 |
| S_1_/S_3_ | *aa* | -0.17 | 0.25 | S_3_/S_6_ | *aa* | -1.00 | -1.95 |
|  | *ad* | -2.67 | -3.29 |  | *ad* | -2.42 | -2.56 |
|  | *da* | -2.00 | -1.60 |  | *da* | -0.83 | -1.70 |
|  | *dd* | -5.00 | -7.98 |  | *dd* | -5.00 | -5.73 |
| S_1_/S_4_ | *aa* | -1.83 | -2.29 | S_4_/S_6_ | *aa* | 0.75 | 1.04 |
|  | *ad* | 3.25 | 4.85 |  | *ad* | -0.67 | -0.04 |
|  | *da* | 5.75 | 7.79 |  | *da* | 2.67 | 4.64 |
|  | *dd* | -0.33 | 1.35 |  | *dd* | -0.67 | 0.64 |
